# Thyroid Storm, Rhabdomyolysis, and Pulmonary Embolism: An Unusual Triad Case Report

**DOI:** 10.5811/cpcem.2020.7.47774

**Published:** 2020-09-03

**Authors:** Montane B. Silverman, Jesse Wray, Rachel E. Bridwell, Amber Cibrario

**Affiliations:** * Uniformed Services University F. Edward Hebert School of Medicine, Bethesda, Maryland; † Brooke Army Medical Center, Department of Emergency Medicine, Fort Sam Houston, Texas

**Keywords:** Thyroid storm, rhabdomyolysis, pulmonary embolism, case report

## Abstract

**Introduction:**

Thyroid storm is a medical emergency associated with significant mortality. Hyperthyroid states have been associated with hypercoagulability as well as rhabdomyolysis. However, the pathophysiology of this association remains under investigation.

**Case Report:**

A 62-year-old male patient presented to the emergency department with weakness and was found to have thyroid storm with concurrent submassive pulmonary embolisms and rhabdomyolysis. To our knowledge, this is the first reported presentation of this triad.

**Conclusion:**

This case highlights the potentially difficult diagnosis and management of thyroid storm, as well as associated life-threatening complications, including venous thromboemboli and rhabdomyolysis.

## INTRODUCTION

Hyperthyroid states exist on a spectrum of disease severity; this spectrum ranges from subclinical hyperthyroidism to clinical manifestations of this hormone derangement, or thyrotoxicosis, to the most severe form with multisystem involvement and evidence of systemic decompensation – thyroid storm.^1^ Thyroid storm most commonly affects female patients, with an associated mortality rate between 8–25%, and those over 60 years of age have worse outcomes.^1^ This life-threatening condition characterized by adrenergic overactivity can be secondary to a myriad of etiologies and is often triggered by stress-inducing states such as surgery, trauma, or infection.^1^

Classically, thyroid storm is a clinical diagnosis of thyrotoxicosis with multisystem deterioration. This diagnosis is augmented by clinical scoring systems such as the Burch-Wartofsky Point Scale (BWPS). BWPS uses a point scale to assign patients into three categories suggesting their likelihood of progressing to fulminant thyroid storm. The scale incorporates temperature, central nervous system effects, gastrointestinal and hepatic dysfunction, heart rate, evidence of congestive heart failure, and the presence or absence of atrial fibrillation as well as a precipitating event (Table).^1^,^2^

Previous literature has demonstrated that hyperthyroid states have been associated with hypercoagulability leading to venous thromboembolic (VTE) events such as cerebral thrombosis, deep vein thrombosis (DVT), and pulmonary embolism (PE).^3^–^10^ Additionally, multiple cases report rhabdomyolysis as a sequela of hyperthyroid states.^11^–^15^ To our knowledge, this is the first reported case of a patient with thyroid storm complicated by both rhabdomyolysis and submassive PE at time of presentation to the emergency department (ED).

## CASE REPORT

A 62-year-old male with past medical history of prior cerebral vascular accident and coronary artery disease presented to the ED by emergency medical services with three days of extremity weakness, fatigue, and imbalance. The patient’s spouse reported that he experienced lethargy, myalgias, decreased appetite, subjective fevers, and poor sleep over this time. One day prior to presentation, the patient fell secondary to generalized weakness without a loss of consciousness, which ultimately prompted presentation for medical evaluation, after lying on the floor for approximately four hours.

CPC-EM Capsule

What do we already know about this clinical entity?

*Thyroid storm is a medical emergency with significant mortality. Hyperthyroid states have been associated with both hypercoagulability and rhabdomyolysis*.

What makes this presentation of disease reportable?

*To our knowledge this is the first reported case of a patient presenting to the ED with thyroid storm, rhabdomyolysis, and a pulmonary embolism*.

What is the major learning point?

*Thyroid storm can be associated with life-threatening complications such as rhabdomyolysis and pulmonary embolism*.

How might this improve emergency medicine practice?

*By having suspicion for additional life-threatening complications, the emergency provider can improve the management of thyroid storm*.

On presentation to the ED, vital signs included heart rate of 121 beats per minute (bpm), blood pressure of 130/103 millimeters of mercury (mm Hg), respiratory rate of 20 breaths per minute with a saturation of 95% on room air, and oral temperature of 101.5 degrees Fahrenheit. Physical examination was notable for tachycardia, diffuse abdominal pain without evidence of peritonitis, a resting tremor, and mild confusion.

Electrocardiogram was notable for sinus tachycardia to 121 bpm without evidence of ischemia or right heart strain. Serum studies demonstrated a thyroid stimulating hormone level of 0.007 microinternational units (uIU) per microliter (uL) (normal 0.27–5.00 uIU/uL), free T4 (FT4) of 2.94 nanograms per deciliter (ng/dL) (normal 0.6–1.8 ng/dL), elevation of aminotransferases with an aspartate aminotransferase of 232 units (U) per liter (L) (normal 5–40 U/L) and an alanine aminotransferase of 78 U/L (normal 4–41 U/L), creatinine of 1.23 milligrams (mg)/dL (normal 0.67–1.17 mg/dL), troponin of 0.081 ng/mL (normal <0.03 ng/mL), and B type natriuretic peptide was elevated to 2852 picograms (pg)/mL (normal <900 pg/mL). Creatinine kinase (CK) was also elevated to 32,290 IU/L (normal 24–170 IU/L), and urinalysis demonstrated large amounts of blood with only two red blood cells/high powered field (normal 0–3). A computed tomography (CT) of the abdomen and pelvis showed no abnormalities, although a PE was noted in the right lower lobe of the lung (Image), which was confirmed with a subsequent CT pulmonary angiogram along with an additional right upper lobe PE.

Based on the patient’s presentation with abnormal thyroid function, BWPS score was calculated as 50, which is highly suggestive of thyroid storm. He was started on propranolol, propylthiouracil, Lugol’s iodine solution, and hydrocortisone with improvement of his vital signs and symptoms over the following several days. Therapeutic heparin was initiated for multiple submassive pulmonary emboli, with resolution of elevated troponin. Anticoagulation was transitioned to apixaban for continued outpatient treatment.

Additionally, the patient was diagnosed with rhabdomyolysis in the setting of myalgias, elevated CK well above five times the upper limit of normal, acute kidney injury, and myoglobinuria. He received intravenous fluids with a urine output goal of 200–300 mL/hour as well as allopurinol due to hyperuricemia. CK and uric acid levels were trended throughout hospitalization, which progressively improved prior to discharge. He was ultimately discharged after an otherwise uneventful hospitalization.

## DISCUSSION

Both hyperthyroidism and hypothyroidism precipitate a variety of physiologic derangements, including hypercoagulable and hypocoagulable states. The effects of these changes in coagulability range from subclinical to fatal coagulative events.^3^ There is no current consensus on the pathophysiology of this hypercoagulability as numerous studies have shown a wide effect of hyperthyroidism on platelets, coagulations factors, and von Willebrand factor.^4^,^5^ However, a variety of data demonstrates that increased levels of thyroid hormone alter the coagulation-fibrinolysis equilibrium and this increase in hormone level is an independent risk factor for VTE.^3^,^5^–^6^

A clear dose response of FT4 in both PE and DVT has been established.^7^ While this association could be attributed to a non-thyroidal illness syndrome, in which a decrease in peripheral conversion of T4 to T3 is seen, this has been generally dismissed in the literature based on increased levels of T3 in cases of VTE associated with hyperthyroid disease.^7^ Although thyrotoxicosis and thyroid storm precipitate both arterial and venous thrombosis, the majority of the literature on hypercoagulability focuses on endocrine management rather than that of VTEs.^8^,^9^ The management, prophylaxis, and occurrence of VTE in patients with thyroid storm remain a site of investigation, although quelling the precipitating event and preventing VTE propagation are the mainstay of treatment.

Despite the current body of literature reflecting hypothyroidism as a risk factor for the development of rhabdomyolysis, there are very limited data that have demonstrated an association between hyperthyroid states and rhabdomyolysis.^10^–^12^ These limited cases occurred both with and without significant exertion.^13^ While the pathophysiology of hyperthyroid-induced rhabdomyolysis has not been established, it is hypothesized that the increased metabolic rate exhausts muscular substrates and energy stores.^11^,^12^ By definition, rhabdomyolysis requires elevated CK levels and myalgias, which differentiates it from the other neuromuscular-involved pathologies of hyperthyroidism such as thyrotoxic myopathy, thyrotoxic periodic paralysis, and thyroid ophthalmopathy.^11^,^14^ In this case, the patient sustained a fall resulting in multiple hours on the ground, a common precipitant of rhabdomyolysis. However, the patient had been experiencing generalized symptoms including myalgias and weakness prior to the fall. Although it is probable that the fall contributed to the patient developing rhabdomyolysis, hyperthyroidism likely also contributed either directly via increased metabolism or indirectly via generalized weakness resulting in a fall.

Much like the above case, a similar presentation described a patient with thyroid storm who subsequently developed rhabdomyolysis as well as a DVT and subclinical PE during her hospitalization.^15^ Although that prior case established these three diagnostic entities occurring in a single patient, our case is the first report of a patient with these pathologies diagnosed both simultaneously and acutely on presentation in the ED. A review of the current literature did not find independent correlation between VTE disease and rhabdomyolysis. Without independent correlation between these two entities, hyperthyroid pathology is the most likely underlying etiology related to both the PE and rhabdomyolysis. The authors hypothesize that this patient developed a venous thrombosis with embolization to the pulmonary arteries as a consequence of the hyperthyroid condition. Additionally, rhabdomyolysis was likely from the hypermetabolic state induced by the thyroid storm itself, the fall sustained secondary to weakness from thyroid storm, or a combination of these two factors.

## CONCLUSION

With the established severe morbidity and mortality of thyroid storm, it is crucial that emergency physicians quickly recognize and manage this pathology. Diagnostic tools such as the BWPS can aid in early identification. Emergency physicians must also be aware of conditions associated with thyroid storm that can further compound morbidity and mortality. While the pathophysiology of thyroid storm and its association with hypercoagulability and rhabdomyolysis remains nebulous, their association can generate critically ill patients. The above case demonstrates that both venous thromboembolic disease and rhabdomyolysis can occur acutely with thyroid storm, reflecting the heightened suspicion that physicians should maintain for the resulting rhabdomyolysis and VTE in patients presenting with thyroid storm.

## Figures and Tables

**Image:**
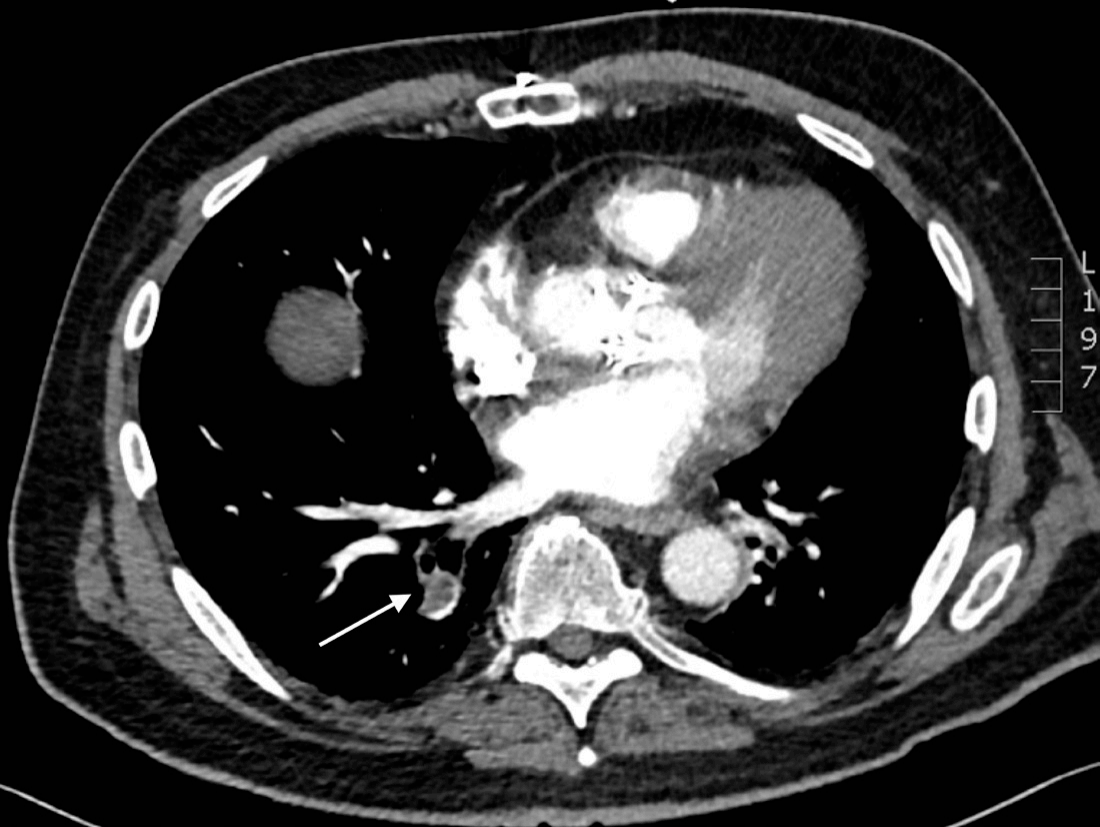
Computed tomography of the chest with contrast demonstrating a right lower lobe pulmonary embolism (arrow).

**Table:** The Burch-Wartofsky Point Scale is used as a clinical scoring system for hyperthyroid states.

Criteria	Point value
Temperature (°F)	
<99	0
99–99.9	5
100–100.9	10
101–101.9	15
102–102.9	20
103–103.9	25
>104.0	30
Central Nervous System Symptoms	
None	0
Mild (agitation)	10
Moderate delirium, psychosis, lethargy)	20
Severe (seizure, coma)	30
Gastrointestinal-hepatic Symptoms	
None	0
Moderate (diarrhea, nausea/vomiting, abdominal pain)	10
Severe (unexplained jaundice)	20
Cardiovascular dysfunction (beats/min)	
<90	0
90–109	5
110–119	10
120–129	15
130–139	20
>140	25
Atrial fibrillation	
Absent	0
Present	10
Heart failure	
None	0
Mild (pedal edema)	5
Moderate (bibasilar rales)	10
Severe (pulmonary edema)	15
Precipitant history	
Absent	0
Present	10
Total Score	
Thyroid Storm Unlikely	<25
Impending Storm	25–45
Highly Suggestive of Thyroid Storm	>45

*F*, Fahrenheit; *min*, minutes.
